# Innovative polymer engineering for the investigation of electrochemical properties and biosensing ability

**DOI:** 10.55730/1300-0527.3611

**Published:** 2023-09-28

**Authors:** Sıla Can OSMANOĞULLARI, Saniye SÖYLEMEZ, Oğuzhan KARAKURT, Serife ÖZDEMİR HACIOĞLU, Ali ÇIRPAN, Levent TOPPARE

**Affiliations:** 1 Department of Chemistry, Faculty of Science, Karadeniz Technical University, Trabzon, Turkiye; 2 Department of Biomedical Engineering, Faculty of Engineering, Necmettin Erbakan University, Konya, Turkiye; 3 Department of Chemistry, Faculty of Arts and Science, Middle East Technical University, Ankara, Turkiye; 4 Department of Basic Sciences of Engineering, Faculty of Engineering and Natural Sciences, İskenderun Technical University, Hatay, Turkiye; 5 Department of Polymer Science and Technology, Middle East Technical University, Ankara, Turkiye; 6 Center for Solar Energy Research and Application (GÜNAM), Middle East Technical University, Ankara, Turkiye; 7 Department of Micro and Nanotechnology, Middle East Technical University, Ankara, Turkiye; 8 Department of Biotechnology, Middle East Technical University, Ankara, Turkiye

**Keywords:** Fluorine-substituted benzothiadiazole and indole moieties, conjugated polymers, copolymerization, uses in optoelectronics and biosensing, glucose biosensor

## Abstract

Subtle engineering for the generation of a biosensor from a conjugated polymer with the inclusion of fluorine-substituted benzothiadiazole and indole moieties is reported. The engineering includes the electrochemical copolymerization of the indole-6-carboxylic acid (**M1**) and 5-fluoro-4,7-bis(4-hexylthiophen-2-yl)benzo[*c*][1,2,5]thiadiazole (**M2**) on the indium tin oxide and graphite electrode surfaces for the investigation of both their electrochemical properties and biosensing abilities with their copolymer counterparts. The intermediates and final conjugated polymers, Poly(M1) **[P-In6C]**, Poly(M2) **[P-FBTz]**, and copoly(M1 and M2) **[P-In6CFBTz]**, were entirely characterized by ^1^H NMR, ^13^C NMR, CV, UV-Vis-NIR spectrophotometry, and SEM techniques. HOMO energy levels of electrochemically obtained polymers were calculated from the oxidation onsets in anodic scans as −4.78 eV, −5.23 eV, and −4.89 eV, and optical bandgap (Eg^op^) values were calculated from the onset of the lowest-energy π–π* transitions as 2.26 eV, 1.43 eV, and 1.59 eV for **P-In6C**, **P-FBTz**, and **P-In6CFBTz**, respectively. By incorporation of fluorine-substituted benzothiadiazole (**M2**) into the polymer backbone by electrochemical copolymerization, the poor electrochemical properties of **P-In6C** were remarkably improved. The polymer **P-In6CFBTz** demonstrated striking electrochemical properties such as a lower optical band gap, red-shifted absorption, multielectrochromic behavior, a lower switching time, and higher optical contrast. Overall, the newly developed copolymer, which combined the features of each monomer, showed superior electrochemical properties and was tested as a glucose-sensing framework, offering a low detection limit (0.011 mM) and a wide linear range (0.05–0.75 mM) with high sensitivity (44.056 μA mM^−^^1^ cm^−^^2^).

## 1. Introduction

There has been a significant search for viable architectures for numerous uses in recent decades. The diversity of conjugated polymers (CPs) has been regarded as a possible platform for a range of applications. Due to their superior features, such as good film-making ability, stability, ease of tunability of electronic properties, and biocompatibility, they have been extensively utilized in various applications [1]. Organic photovoltaics [2], organic light-emitting diodes [3], electrochromic devices [4], capacitors [5], and biosensors [6] are at the forefront of the application areas of CPs [1]. Due to the ease of chemical tunability of CPs, their electrical conductivity can be adjusted from insulator to metal. At the same time, their mechanical properties can be tuned to a wide extent in pursuit of the desired application [7–9]. Organic electronic sensor technology, i.e., CP-based biosensors, is made possible by combining the host abilities and electrical properties of CPs with the biomolecules’ sharply specific detecting abilities. These remarkable abilities of CP-based biosensors come from the unique and arrangeable optoelectronic properties of CPs [10–12]. In addition, the usage of CPs is favorable because they mirror the conditions in which biomolecules naturally arise while also allowing structural and electrical modifications to attain the best features.

Since the human system must manage blood sugar levels, having too much or too little can cause serious and even deadly issues, such as an increased risk of heart disease, kidney failure, or blindness because of diabetes mellitus [13]. Therefore, the detection of sugar levels in the blood is very crucial. Among the traditional glucose detection methods, electrochemical methods have the advantages of being cost-effective, sensitive, selective, and easy to operate [14]. In the literature, there are numerous studies addressing glucose determination, as summarized in Table 1. For providing the best features for sensing platforms, CPs have an excellent reputation for establishing biomolecular-based scaffolding for the advancement of biosensing materials. Especially with the use of different CPs on biosensor constructs as an immobilization matrix, this may have greater advantages than using the polymers separately. In other words, one of the most practical processes for altering different characteristics of polymers is copolymerization [15]. Copolymer-based biosensors have also been thoroughly investigated in the past [16].

**Scheme 1:**
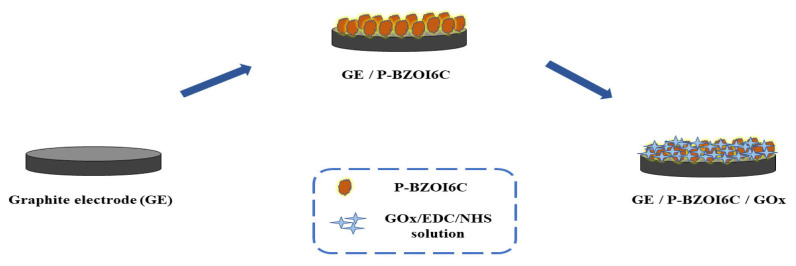
Schematic representation of the designed biosensor.

In light of the given information, three different conjugated polymers, referred to as **P-In6C**, **P-FBTz**, and **P-In6CFBTz**, were synthesized in this study by the electropolymerization method (Scheme 1). For this purpose, indole-6-carboxylic acid (**M1**) and 5-fluoro-4,7-bis(4-hexylthiophen-2-yl)benzo[*c*][1,2,5]thiadiazole (**M2**) were selected as monomers of the three target polymers. Benzothiadiazole (**BTz**) is one of the famous acceptor moieties used for conjugate polymer synthesis due to two unique properties: its striking electron-acceptor feature and its planarity. In this study, the fluorine atom was substituted into the **BTz** core to lower the HOMO levels of the constructed polymers. Thus, it was aimed to reduce the band gap of the constructed polymers to maximize the absorption and optoelectronic abilities. Another specialty of the fluorine atom is its ability to generate noncovalent interactions with neighboring atoms (F-H/S/F/N), which can lead to strong π–π interactions. These interactions may enhance the crystallinity of the morphology and charge carrier mobility, which may boost the optoelectronic properties of the conjugated polymers [17–19]. In addition, the fluorine atoms on the polymer backbone serve as an efficient, highly sensitive, and fast response biosensor system [20]. Lastly, n-hexylthiophene was incorporated to the **BTz** core. As reported in the literature, the insertion of heteroaromatic rings such as selenophene, thiophene, or thienothiophene notably changes the optoelectronic properties of polymers [21,22]. Thus, to boost the solubility and π-conjugation length of the polymer, n-hexylthiophene was used as a π-bridge in the polymer backbone. In recent studies, indole-6-carboxylic acid (**M1**) has attracted much attention due to its distinct benefits, including its strong redox activity and stability as well as its reasonably good thermal stability [23] and high-performance biosensing systems [24].

## 2. Experimental

### 2.1. Materials and equipment

For the synthesis of the corresponding monomer, all reagents and starting materials were commercially obtained and used without any purification. Standardized methods were used to obtain the dried solvents that were used during experiments. Indole-6-carboxylic acid (**M1**) was commercially supplied by Sigma-Aldrich (St. Louis, MO, USA). All experiments were conducted under an inert atmosphere unless otherwise noted. For the purification of crude materials, column chromatography was performed with silica gel 60 (particle size: 0.063–0.200 nm; Merck, Darmstadt, Germany) as the stationary phase. ^1^H NMR chemical shifts were given in ppm downfield from tetramethylsilane (TMS), recorded in CDCl_3_ solvent on a Bruker Spectrospin Avance DPX-400 Spectrometer (Bruker, Billerica, MA, USA). β-D-glucose and glucose oxidase (GOx, β-D-glucose: oxygen 1-oxidoreductase, EC1.1.3.4, 17300 units/g solid from *Aspergillus niger*) were obtained from Sigma Aldrich. Na_2_HPO_4_ (0.025 M) and NaH_2_PO_4_ (0.025 M) from Fisher Scientific Company (Hampton, NH, USA) were used to prepare 50 mM phosphate buffer solution (PBS). Glucose solution (0.1 M, pH 7.0) was used as a substrate. During enzyme fixation, a 1% glutaraldehyde solution in a pH 7.0 buffer was employed.

For electrochemical polymerization studies, a three-electrode cell configuration was used, including an indium tin oxide (ITO)-coated glass slide (12 Ω/cm^2^) as the working electrode, a platinum wire as the auxiliary electrode, and Ag wire as the pseudo electrode, using a Gamry Reference 600 potentiostat (Gamry, Warminster, PA, USA). With the help of a Solartron 1285 potentiostat (Solartron, Meerbusch, Germany) and a Varian Cary 5000 UV-Vis-NIR spectrophotometer (Agilent Technologies, Santa Clara, CA, USA), spectroelectrochemistry and kinetic characterizations of the polymer sheet were carried out.

All chronoamperometric studies were conducted using a potentiostat (PalmSens, Houten, the Netherlands) with a conventional three-electrode setup (the same configuration was used in electrochemical studies without a graphite electrode as the working electrode). Three-measurement averages with standard deviations served as the data for the amperometric investigations. All tests were carried out at ambient temperatures. The information was presented as the average of three observations and standard deviations were noted as ±SD. The reaction compartment utilized in each amperometric survey contained 10 mL of acetate buffer solution (ABS) at pH 4.5 and was kept at room temperature with moderate stirring and a constant potential of −0.7 V. Following every test, the buffer solution was refilled. After the baseline current reached equilibrium, a certain dose of glucose was injected into the reaction medium, and equilibrium was then restored. For capturing biosensor responses, the most recent modification was used. Surface characterizations of the modified electrodes were evaluated by scanning electron microscopy (SEM) (JSM-6400 model, JEOL, Tokyo, Japan).

### 2.2. Biosensor preparation

Each graphite rod underwent emery paper polishing and rinsing with distilled water before the electropolymerization process. Homopolymerization was achieved within the only corresponding monomer solutions under the given experimental conditions (**P-In6C** and **P-FBTz)**. For the copolymerization process to obtain **P-In6CFBTz**, the **M1** (9.7 mg in 6 mL of ACN) and **M2** (7.25 mg in 1 mL of DCM + 5 mL of ACN) monomers were mixed in a 1:5 volume ratio with 0.1 M NaClO_4_/LiClO_4_ (1:1) as the supporting electrolytes. Cyclic voltammetry (CV) was used for the electropolymerization in the potential range between −0.3 V and 0.3 V at a scan rate of 100 mV/s for 10 cycles. Each electrode was washed with distilled water to eliminate unwanted residues following the completion of the copolymerization process. Subsequently, 4 μL of GOx (1.50 mg in 5 μL of PBS, pH 7.0) was immobilized on a copolymer-coated graphite electrode with 3 μL of 1-ethyl-3-(3-dimethylamiopropyl)carbodiimide (EDC) solution (0.4 M in 50 mM PBS, pH 7.0) and 3 μL of N-hydroxysuccinimide (NHS) solution (0.1 M in 50 mM PBS, pH 7.0) as crosslinking agents to reduce enzyme leaching. The electrode was kept at 4 °C overnight after it was left to dry for 2 h at ambient conditions (Scheme 1). Electrodes were rinsed with distilled water before the measurements in order to remove any unattached enzyme molecules.

### 2.3. Synthesis of 5-fluoro-4,7-bis(4-hexylthiophen-2-yl)benzo[c][1,2,5]thiadiazole (M2)

**Scheme 2:**
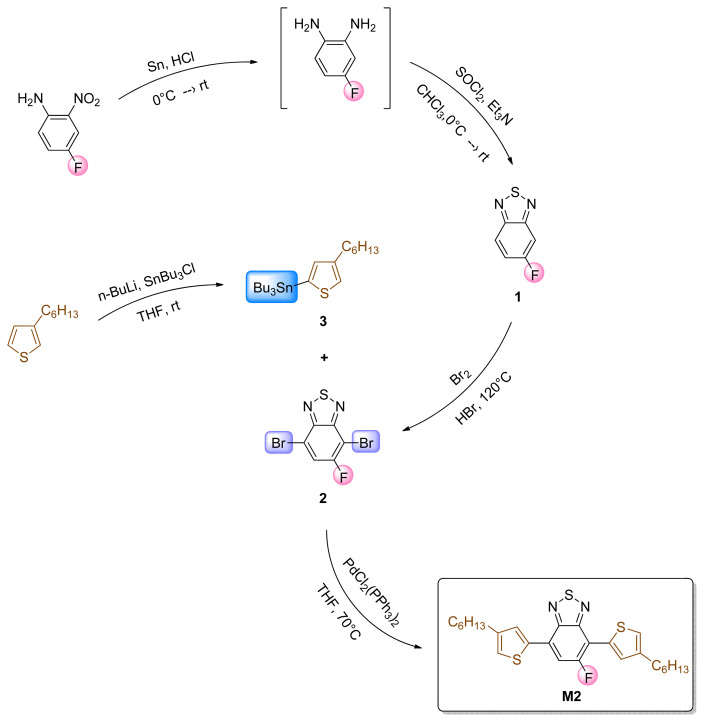
Practical synthetic pathway for the synthesis of the 3-hexylthiophene-modified benzothiazole-based monomer (**M2**).

5-Fluorobenzo[c][1,2,5]thiadiazole(**1**),4,7-dibromo-5-fluorobenzo[c][1,2,5]thiadiazole (**2**), tributyl(4-hexylthiophen-2-yl)stannane (**3**) and 5-fluoro-4,7-bis(4-hexylthiophen-2-yl)benzo[*c*][1,2,5]thiadiazole (**M2**) were synthesized according to previously reported methods in the literature [19,25,26]. First, a reduction reaction was performed to reduce the nitro group to an amine group by using tin and hydrochloric acid, and the intermediate product (4-fluorobenzene-1,2-diamine) was obtained [27]. To synthesize compound **1**, the previously obtained intermediate was treated with thionyl chloride and the target compound was successfully obtained. After that, a bromination reaction with molecular bromine (Br_2_) and hydrobromic acid was performed and compound **2** was successfully synthesized. Compound **3** was synthesized via treatment of 3-hexylthiophene with n-butyl lithium and 3-butyl tin chloride, respectively. Finally, the target compound (**M2**) was synthesized by Stille cross-coupling reaction with molecules **2** and **3** in the presence of a palladium catalyst. The reactions and structures of the compounds are described in detail in Scheme 2. **M2**: ^1^H NMR (400 MHz, CDCl_3_) δ 8.09 (s, 2H), 7.98 (s, 2H), 7.73 (d, *J* = 13.0 Hz, 2H), 7.15 (s, 2H), 7.09 (s, 2H), 2.70 (t, *J* = 7.8 Hz, 4H), 1.74–1.65 (m, 4H), 1.43–1.28 (m, 12H), 0.90 (t, *J* = 6.9 Hz, 6H); ^13^C NMR (100 MHz, CDCl_3_) δ 160.0 (s), 157.5 (s), 153.33 (d, *J* = 11.0 Hz), 149.7 (s), 144.4 (s), 143.4 (s), 137.5 (s), 132.1 (d, *J* = 5.6 Hz), 131.4 (d, *J* = 7.9 Hz), 126.2 (d, *J* = 716 Hz), 125.6 (d, *J* = 11.3 Hz), 122.8 (d, *J* = 6.9 Hz), 116.6 (d, *J* = 32.4 Hz), 111.10 (d, *J* = 15.3 Hz), 31.59 (d, *J* = 2.1 Hz), 30.42 (t, *J* = 6.3 Hz), 28.92 (d, *J* = 2.5 Hz), 22.53 (s), 14.01 (s), 0.91 (s).

## 3. Results and discussion

### 3.1. Electrochemical studies

**Scheme 3:**
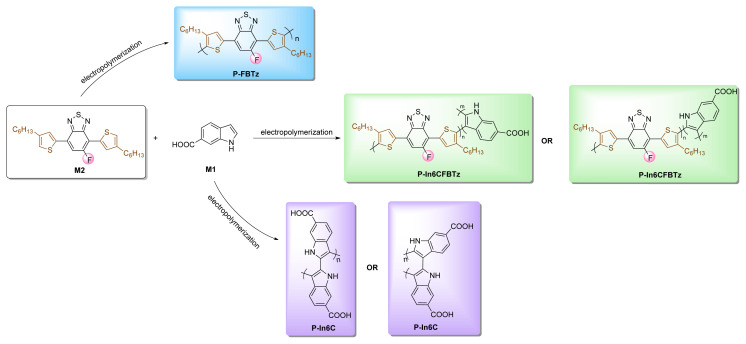
Practical synthetic pathways for the synthesis of target polymers **P-FBTz**, **P-In6CFBTz**, and **P-In6C**.

CV is a multipurpose and functional technique. It is widely preferred for electroactivity determination for compounds and quantitative analyses such as HOMO-LUMO energy levels and oxidation/reduction potentials. Furthermore, electrochemical polymerization and copolymerization can be performed via CV using simple chemicals. In this study, due to the aforementioned benefits and simplicity, electrochemical polymerizations of both homopolymers **P-In6C** and **P-FBTz** and the copolymer **P-In6CFBTz** (at a monomer feed ratio of **M1**:**M2**/1:1) were performed via CV. While the electrochemical polymerization of **P-In6C** was performed in a 0.1 M sodium perchlorate-lithium perchlorate (NaClO_4_–LiClO_4_)/acetonitrile (ACN) electrolyte/solvent couple, electrochemical polymerizations of **P-FBTz** and copolymer **P-In6CFBTz** were performed in a 0.1 M NaClO_4_–LiClO_4_/DCM/ACN (10/90, v/v) electrolyte/solvent system. Scheme 3 depicts the synthetic pathways for the synthesis of the target polymers.

**Figure 1:**
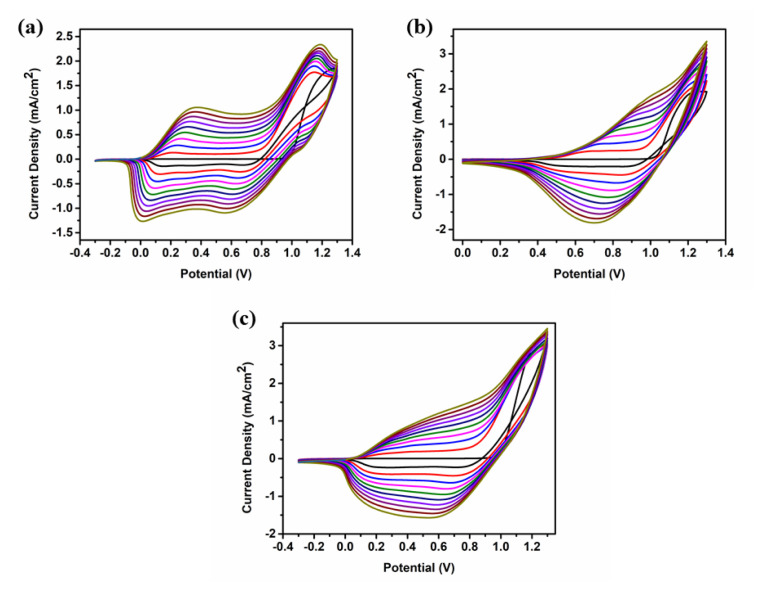
Cyclic voltammograms of (a) **P-In6C**, (b) **P-FBTz**, and (c) **P-In6CFBTz** in 0.1 M NaClO_4_–LiClO_4_/DCM:ACN solution at a scan rate of 100 mV/s.

**Figure 2:**
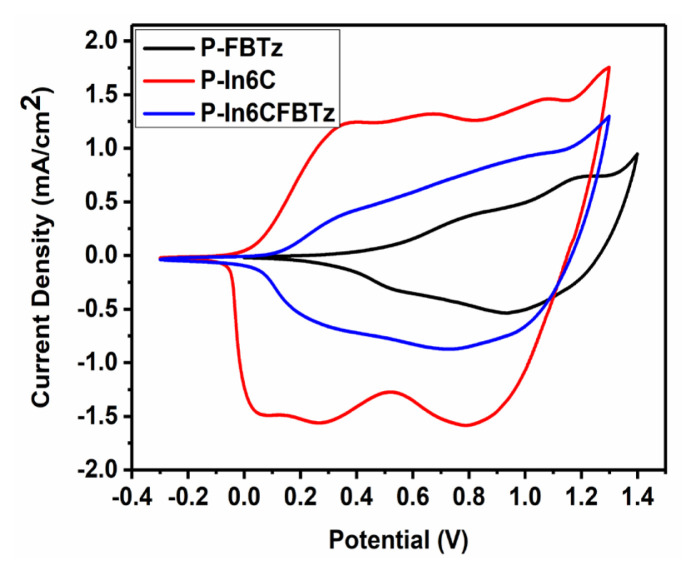
Single-scan cyclic voltammograms of **P-In6C**, **P-FBTz**, and **P-In6CFBTz** in a monomer-free 0.1 M NaClO_4_–LiClO_4_/ACN solution.

The CVs are illustrated in Figure 1 for both the homopolymers and the copolymer. As seen in the first cycle of the CVs, peaks of irreversible monomer oxidation were observed at 1.23 V for **P-In6C**, 1.25 V for **P-FBTz**, and 1.25 V for **P-In6CFBTz**. These similar oxidation potentials of **M1** and **M2** show that these co-monomers are good candidates for copolymerization studies. In addition, the increase in the current density as the cycle number increased proved the success of the electrochemical polymerization and copolymerization. After the successive electropolymerization, polymer-coated ITO electrodes were washed with ACN to remove the unreacted monomer and oligomer, and single-scan CVs were recorded in a 0.1 M NaClO_4_–LiClO_4_/ACN solution (Figure 2). The potential range of the CV scan of **P-IN6C** and **P-In6CFBTz** was between −0.3 V and 1.3 V and that of P-FBTz was between 0.0 V and 1.3 V. The p-type doping behavior with reversible oxidation potentials was observed in both the homopolymers and copolymers at 0.37 V/0.67 V/1.07 V for **P-In6C**, 0.87 V/1.21 V for **P-FBTz**, and 0.37 V/1.05 V for **P-In6CFBTz**. The HOMO energy levels of the electrochemically obtained polymers were calculated from the oxidation onsets in anodic scans as −4.78 eV, −5.23 eV, and −4.89 eV for **P-In6C**, **P-FBTz**, and **P-In6CFBTz**, respectively.

### 3.2. Spectroelectrochemical properties

For further characterization, spectroelectrochemical measurements were performed for **P-In6C**, **P-FBTz**, and **P-In6CFBTz** in order to explore the optical properties and changes after the doping processes. Spectroelectrochemical measurements were carried out using a UV-Vis-NIR spectrophotometer integrated with a potentiostat in a monomer-free 0.1 M NaClO_4_–LiClO_4_/ACN solution. Initially, all polymers were formed electrochemically as described above on an ITO electrode, and spectroelectrochemical studies were performed via stepwise oxidation of polymer films between −0.5 V and 1.1 V for **P-In6C**, between −0.5 V and 1.2 V for **P-FBTz**, and between −0.5 V and 1.1 V for **P-In6CFBTz**.

**Figure 3:**
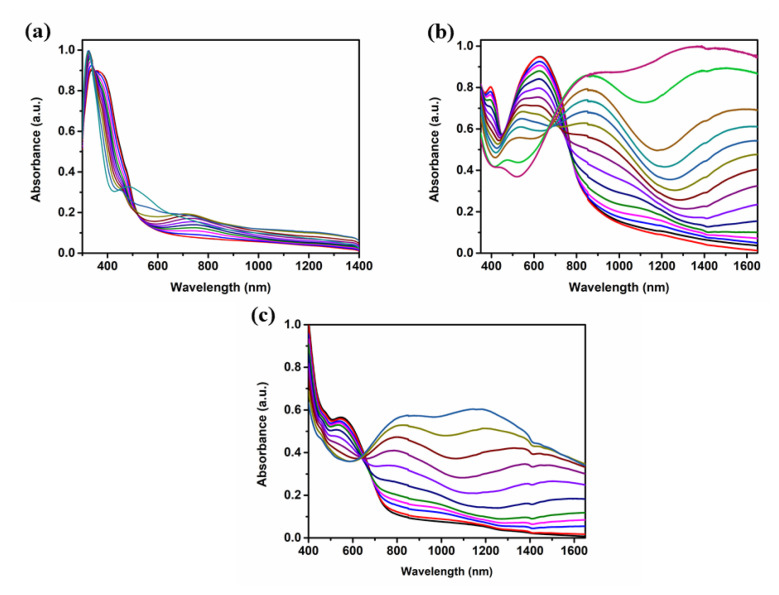
Spectroelectrochemical studies of (a) **P-In6C**, (b) **P-FBTz**, and (c) **P-In6CFBTz** in 0.1 M NaClO_4_–LiClO_4_/ACN electrolyte/solvent couple.

Before stepwise oxidation, in order to remove any trapped charge or ions, −0.5 V fixed potentials were administered and neutral state absorptions were recorded at 371 nm/483 nm for **P-In6C**, 625 nm for **P-FBTz**, and 560 nm for **P-In6CFBTz**. As displayed in Figure 3, during the stepwise oxidation of the polymers, while the neutral-state absorption bands were depleting, new bands known as charge carriers (polarons and bipolarons) appeared around 800 nm and 1200 nm. Optical band gap (E_g_^op^) values are also significant and have an impact on the feasibility for numerous applications. E_g_^op^ values were calculated from the onset of the lowest-energy π–π* transitions and are reported as 2.26 eV, 1.43 eV, and 1.59 eV for **P-In6C**, **P-FBTz**, and **P-In6CFBTz** in Table 2.

**Figure 4:**
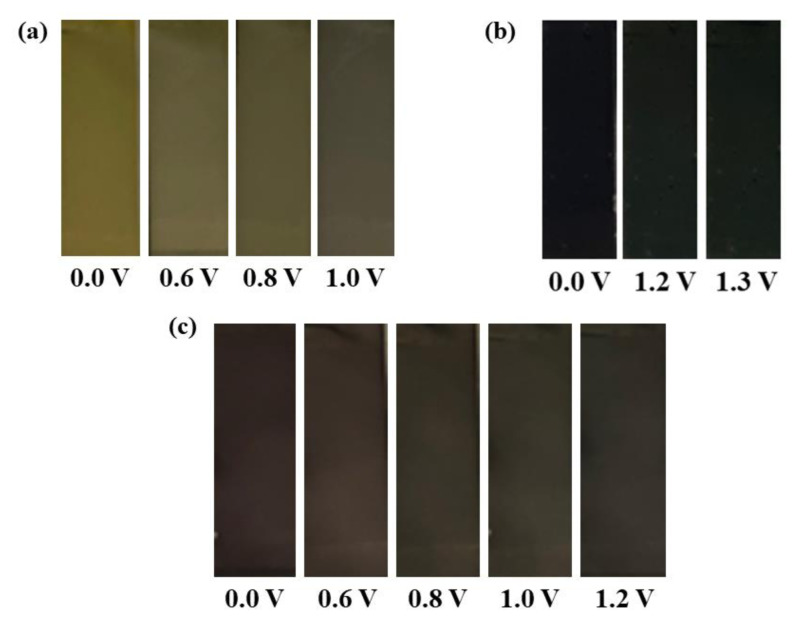
Colors of (a) **P-In6C**, (b) **P-FBTz**, and (c) **P-In6CFBTz** in neutral/oxidized and intermediate states.

In the interest of investigating the electrochromic properties of copolymers and polymers prepared electrochemically, photographs of the polymer films were obtained while applying diverse potentials. Colorimetric determinations were performed at the neutral, oxidized, and different intermediate states and are depicted in Figure 4. **P-In6C** and **P-FBTz** exhibited yellow and blue colors in their neutral states. A considerably red-shifted absorbance with a red-purple copolymer was produced by the copolymerization of **M1** and **M2**. All polymers exhibited multichromic behavior with various shades of gray color in the oxidized states, as shown in Figure 4. While **P-In6C** exhibited a light gray color, **P-FBTz** had a greenish gray color, and **P-In6CFBTz** had a gray color in fully oxidized states.

### 3.3. Electrochromic contrast and switching studies

Investigations of electrochromic contrast and switching are crucial, particularly for the use of the resultant polymers in electrochromic device applications. During kinetic studies, transmittance changes (optical contrast) and switching times were measured while steeping the polymer film between two extreme states (neutral and oxidized states). Optical contrast is the change in percent transmittance values between two extreme states and switching time is the time necessary for one full switch of the polymer between neutral and oxidized states. The maximum UV-Vis-NIR absorbance data were used to determine the wavelengths for electrochromic contrast and switching investigations. All polymers were produced electrochemically, as stated previously, and electrochromic contrast and switching investigations were conducted in a monomer-free 0.1 M NaClO_4_–LiClO_4_/ACN solution while varying the applied potentials between neutral and oxidized states every 5 s.

**Figure 5:**
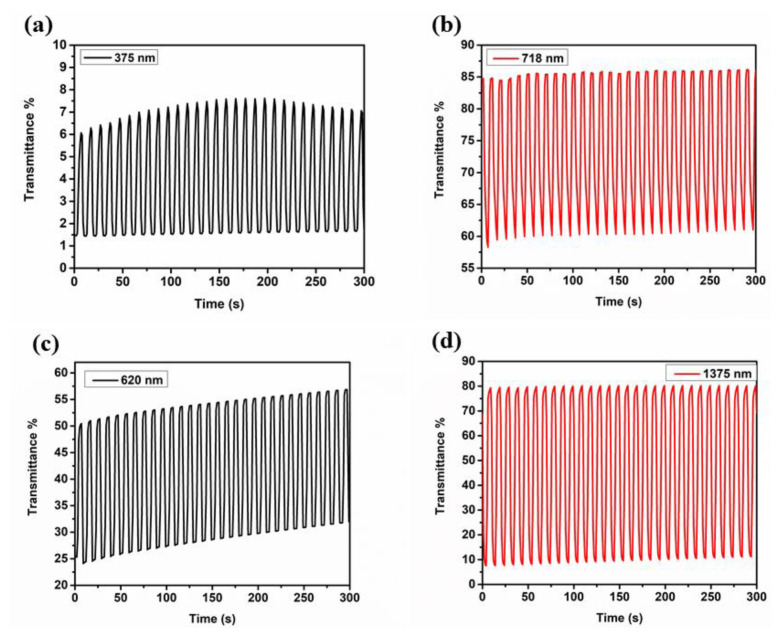
Optical transmittance changes (a, b) for **P-In6C** at 375 nm and 718 nm and (c, d) for **P-FBTz** at 620 nm and 1375 nm with 0.1 M NaClO_4_–LiClO_4_/ACN electrolyte/solvent couple while switching the potentials between neutral and oxidized states.

**Figure 6:**
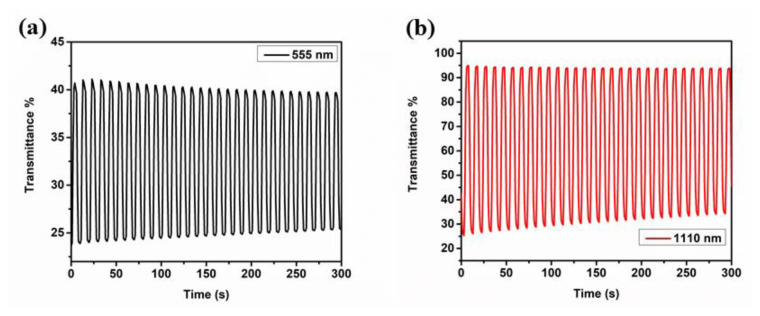
Optical transmittance changes for **P-In6CFBTz** at (a) 555 nm and (b) 1110 nm in 0.1 M NaClO_4_–LiClO_4_/ACN electrolyte/solvent couple while switching the potentials between neutral and oxidized states.

As reported in Table 3 and depicted in Figures 5 and 6, while **P-In6C** showed 6% (at 375 nm) and 27% (at 718 nm) transmittance changes, **P-FBTz** exhibited 27% (at 620 nm) and 72% (at 1375 nm) transmittance changes and, finally, **P-In6CFBTz** revealed 17% (at 555 nm) and 68% (at 1110 nm) optical contrast values. Corresponding switching time values were calculated at the abovementioned wavelengths as 3.3 s and 2.1 s (at 375 nm) and 4.6 s and 1.9 s (at 718 nm) for **P-IN6C**, 1.9 s and 1.9 s (at 620 nm) and 2.6 s and 2.4 s (at 1375 nm) for **P-FBTz**, and 1.7 s and 2.0 s (at 555 nm) and 2.8 s and 2.6 s (at 1110 nm) for **P-In6CFBTz**.

As seen in Tables 2 and 3, the poor electrochemical and electrochromic behaviors of **P-In6C** prevent its applicability in electrochromic device applications. In this study, the electrochromic properties of **P-In6C** were significantly improved via the insertion of the F-substituted benzothiadiazole derivative **M2** into the structure by electrochemical copolymerization. Copolymerization resulted in enhanced electrochromic properties of **P-In6CFBTz**, such as lower optical band gap, red-shifted absorption, multielectrochromic behavior, a lower switching time, and a higher optical contrast.

### 3.4. Biosensor studies

**Figure 7:**
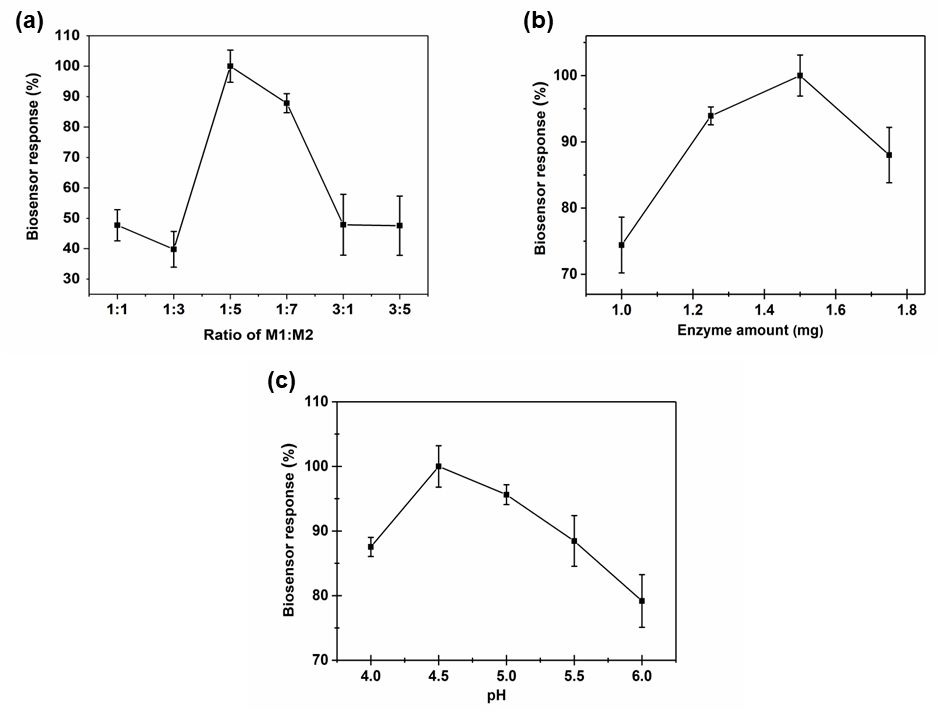
Effect of (a) monomer ratio, (b) enzyme amount, and (c) pH on biosensor response at −0.7 V and 25 °C.

To create a stable and reproducible biosensor system, all factors that have an impact on its effectiveness should be adjusted. The ratio of **M1**:**M2** (v/v), the amount of enzyme, and the pH conditions were all optimized in this manner. First, the effect of the **M1** and **M2** monomer ratios on the biosensor signal with the formation of **P-In6CFBTz** was investigated (Figure 7a). The **M1**:**M2** solution was prepared at ratios of 1:1, 1:3, 1:5, 1:7, 3:1, and 3:5, and **P-In6CFBTz** was coated on the electrode surface with these solutions. After amperometric measurements, the 1:5 (**M1**:**M2**) ratio gave the most ideal sensor response, while a decrease in the biosensor signal was observed when the **M1**:**M2** ratio was increased more. The electrochemical homopolymerization of both the **M1** and **M2** monomers was also carried out and their biosensor abilities were evaluated. However, the obtained amperometric signals were not stable and were insufficient with these homopolymers compared to the sensor obtained with **P-In6CFBTz**, so it was decided to continue the biosensor construction with **P-In6CFBTz**. As a result of the experimental data obtained for all these ratios, 1:5 (**M1**:**M2**) was determined as the ideal ratio for the biosensor. In the next step, the effect of enzyme amount on the copolymer-coated electrochemical biosensor was investigated. Optimizing the amount of enzyme is one of the most important steps because if the amount of enzyme is less than it should be, the biosensor signal may be inadequate. On the contrary, if the amount of enzyme is more than needed, the enzyme may not be adsorbed well on the surface and leaching may occur. In this context, in order to examine the effect of enzyme amount on the biosensor signal, while keeping other variables constant, 1.00 mg (17.30 U), 1.25 mg (21.63 U), 1.50 mg (25.95 U), and 1.75 mg (30.28 U) GOx were dissolved separately in 5 μL of PBS (pH 7.0) and four separate enzyme electrodes were prepared to examine their amperometric signals (Figure 7b). According to the results, 1.5 mg of GOx was selected as the optimum and used for further experiments. Ultimately, the ideal pH variation response for a suitable biosensor was examined. Enzyme molecules are greatly impacted by the pH of the surrounding environment. There is a pH range in which every enzyme operates at its maximum. At different levels of pH, enzyme conformation can alter. With the aim of investigating this, several conditions with a range of pH values from 4.0 to 6.0 were studied. A pH of 4.5 produced the best response (Figure 7c).

**Figure 8:**
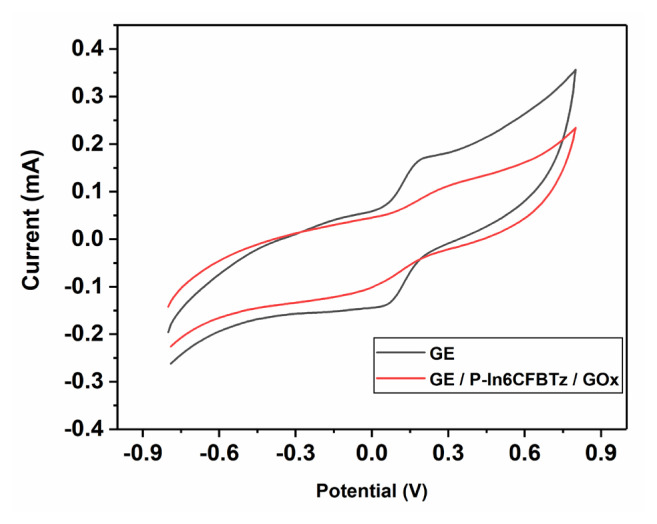
Cyclic voltammograms of GE and GE/**P-In6CFBTz**/GOx in 5.0 mM Fe(CN)_6_^3−/4−^ containing 0.1 M KCl.

The effective surface area was characterized using CV following the electrode modification. A solution incorporating 5.0 mM Fe(CN)_6_^3−/4−^ and 0.1 M KCl in 50.0 mM ABS (pH 4.5) was used for the experiments, which were run at a scan rate of 100.0 mV s^−^^1^ with a potential range of −0.8 V to 0.8 V. Two separate electrodes, including bare GE and GE/**P-In6CFBTz**/Gox, were prepared to show the surface modification after biosensor construction (Figure 8). The Randles–Sevcik equation [28] was utilized for the calculation of the effective surface areas of the GE and GE/**P-In6CFBTz**/GOx electrodes, which have surface areas of 0.146 cm^2^ and 0.096 cm^2^, respectively. The effective surface area decreasing after enzyme immobilization indicated that the enzyme had been successfully attached to the surface.

**Figure 9:**
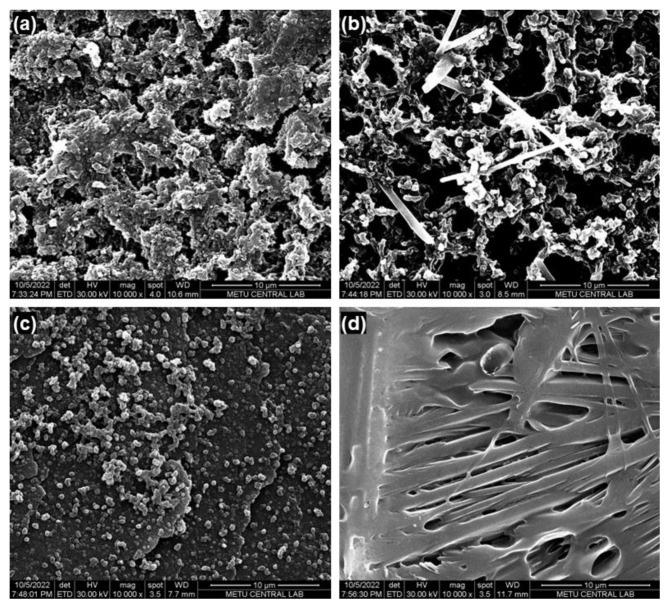
SEM images of (a) GE/**P-In6C**, (b) GE/**P-FBTz**, (c) GE/**P-In6CFBTz**, and (d) GE/**P-In6CFBTz**/GOx under optimized conditions.

Additionally, SEM was used for the surface characterization of the GE/**P-In6C**, GE/**P-FBTz**, GE/**P-In6CFBTz**, and GE/**P-In6CFBTz**/GOx electrodes (Figure 9). The typical cauliflower-like structure was observed for both the GE/**P-In6C** and GE/**P-FBTz** surfaces. After the surface was modified with **P-In6CFBTz**, it was observed that the GE/**P-In6CFBTz** morphology showed GE/**P-In6C** and GE/**P-FBTz** surface characteristics (Figure 9c). However, the GE/**P-In6CFBTz** surface seemed more homogeneous compared to the GE/**P-In6C**- and GE/**P-FBTz**-coated surfaces. The regular surface achieved after the immobilization of GOx onto **P-In6CFBTz**-coated GE showed proper coverage of the enzyme on the **P-In6CFBTz**-coated surface.

**Figure 10:**
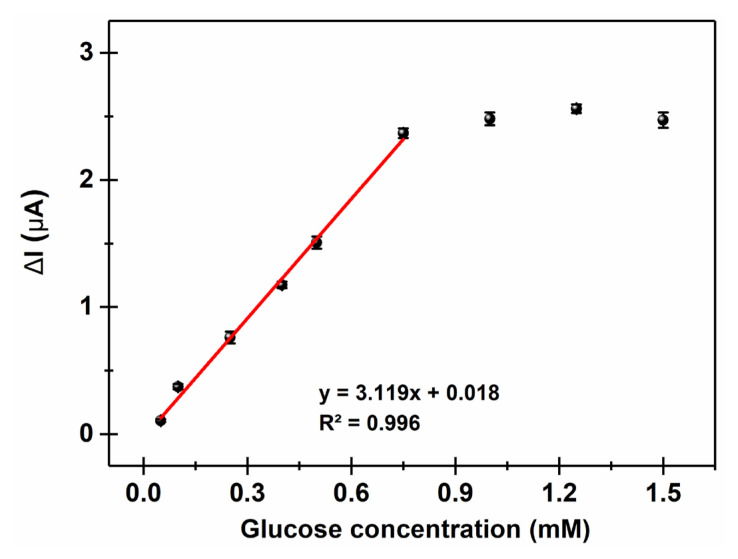
Calibration curve for glucose (in 50 mM ABS, pH 4.5, −0.7 V, 25 °C). Error bars indicate the standard deviations of three measurements.

A calibration curve was created with biosensor responses for varying concentrations of glucose. In Figure 10, the equation y = 3.119x + 0.018 with R^2^ = 0.996 has a linear range between 0.05 mM and 0.75 mM glucose levels in 50 mM ABS (pH 4.5). A saturation point was observed after injecting 0.75 mM glucose. Using S/N = 3, the limit of detection (LOD) and the limit of quantification (LOQ) were calculated as 0.011 mM and 0.037, respectively. The **P-In6CFBTz**-coated enzyme electrode (GE/**P-In6CFBTz**/GOx) has sensitivity of 44.056 μA mM^−^^1^ cm^−^^2^. Compared to the findings in the literature, the constructed biosensor has a superior LOD value. Guler et al. fabricated a glucose biosensor modified with poly(TTP) using GCE and found 0.100 mM as the LOD [29]. Jedrezak et al. used a carbon paste electrode in their study to construct a SiO_2_/lignin-modified glucose electrode and obtained a LOD of 0.145 mM [30]. In order to show the biosensor’s repeatability, the response to a glucose solution of 0.5 mM was assessed 10 times. The outcomes demonstrated that the biosensor’s detection capability after each measurement was almost the same. Values of 5.16% and 0.07 were reported as the relative standard deviation (RSD) and the standard deviation (SD), respectively. Moreover, the *I_max_* value was calculated as 3.98 μA and the *K_Mapp_* value was calculated as 1.77 mM by utilizing the Lineweaver–Burk plot (1/I vs. 1/[S]) [31]. A low *K_Mapp_* value is a sign of high enzymatic activity towards the substrate. Holland et al. employed a glucose biosensor by using maleimide-modified gold nanoparticles with a *K_Mapp_* value of 6.3 mM [32]. In another study of a glucose biosensor, Chen et al. found the *K_Mapp_* value to be 6.5 mM while utilizing a poly(3,4-ethylenedioxythiophene)-modified carbon fiber microelectrode [33].

Furthermore, the effects of various interferences, including those of ascorbic acid, citric acid, and urea, were examined. The GE/**P-In6CFBTz**/GOx biosensor response to 0.5 mM glucose was evaluated in the presence of different interferents with the same concentrations (0.5 mM, adding at the 65th second) in a cell containing 50 mM ABS (pH 4.5) at 25 °C. Although a slight interference effect was observed with ascorbic acid, no significant signal change was observed for the other interferences.

The biosensor’s response was measured for samples of readily accessible coke (0.5 mM glucose as the reference value of C® Coke) in an effort to determine how useful it is in the real world. When the current became constant, coke with 0.5 mM glucose content was inserted into a cell containing 10 mL of 50 mM ABS (pH 4.5). The current change was recorded in the form of amperometric measurements with a constant potential under optimum conditions. From the calibration curve (Figure 10), the glucose content was calculated as 0.494 mM with a recovery rate of 98.8%. These results are very close to the ideal value of 0.50 mM, which shows that the constructed GE/**P-In6CFBTz**/GOx glucose biosensor is suitable for real-world applications.

## 4. Conclusion

In this study, the electrochemical copolymerization of indole-6-carboxylic acid (**M1**) and 5-fluoro-4,7-bis(4-hexylthiophen-2-yl)benzo[*c*][1,2,5]thiadiazole (**M2**) was achieved to investigate both electrochemical properties and biosensing abilities. The electrochemical and optical features of **P-In6CFBTz** were thoroughly studied and it was found to exhibit good electrochemical characteristics. For biosensing engineering, the GE/**P-In6CFBTz**/GOx combination was used to build an effective glucose biosensor. Compared to the homopolymers of the **M1** and **M2** monomers, the sensor system constructed with **P-In6CFBTz** demonstrated an outstanding sensing material and excellent sensor performance. To the best of our knowledge, neither the electrochemical characteristics of the copolymer nor the biolayer of the corresponding polymers for amperometric glucose detection have been previously reported in the literature. Comparing the developed sensor properties to literature examples, it appears to be quite promising. The constructed glucose biosensor exhibits outstanding kinetic parameters with low *K_Mapp_* and LOD values and high sensitivity values. The constructed biosensor’s real-sample applicability was observed with satisfactory results.

## Figures and Tables

**Table 1:** ePrevious studies of glucose biosensors.

Electrode modification	Linear Range (mM)	LOD (mM)	Sensitivity (μA mM^−1^ cm^−2^)	Application	Reference
Chitosan/GOx/LIGE	0.0–8.0	0.431	43.15	NR	[34]
Alginate-Cryogel/NiFe_2_O_4_/GOx/GCE	1.0–5.0	0.320	NR	NR	[35]
Au/ZnO/GOx-Fc/MWCNT	0.0–12.0	0.250	30.00	Human blood	[36]
PANI-TT-GOx/GCE	0.01–1.0	0.010	24.29	Human urine	[37]
TiO_2_/SrTiO_3_/PDA/GOx	0.0–32.0	0.026	5.37	Human serum	[38]
GO/Co/Chitosan/AuE	1.0–15.0	2.700	0.14	Beverage	[39]
**P-In6CFBTz/GOx/GE**	0.05–0.75	0.011	44.056	C^®^ Coke	This work

NR: Not reported.

**Table 2:** Electrochemical and spectroelectrochemical properties of the polymers.

	E_m_^ox^	E_p-doping_ (V)	E_p-doping_^onset^ (V)	HOMO (eV)	LUMO (eV)	λ_max_ (nm)	λ_max_^onset^(nm)	E_g_^op^ (eV)
**P-In6C**	1.23	0.37 V/0.67 V/1.07 V	0.03 V	–4.78	–2.52	371 nm/483 nm	549 nm	2.26
**P-FBTz**	1.25	0.87 V/1.21 V	0.48 V	–5.23	–3.80	625 nm	867 nm	1.43
**P-In6CFBTz**	1.25	0.37 V/1.05 V	0.14 V	–4.89	–3.30	560 nm	780 nm	1.59

**Table 3:** Summary of electrochromic contrast and switching studies.

	Wavelength (nm)	Optical contrast (%)	Switching time (reduction) (s)	Switching time (oxidation) (s)
**P-In6C**	375 nm	6%	3.3 s	2.1 s
	718 nm	27%	4.6 s	1.9 s
**P-FBTz**	620 nm	27%	1.9 s	1.9 s
	1375 nm	72%	2.6 s	2.4 s
**P-In6CFBTz**	555 nm	17%	1.7 s	2.0 s
	1110 nm	68%	2.8 s	2.6 s
